# Intercellular transmission of alpha-synuclein

**DOI:** 10.3389/fnmol.2024.1470171

**Published:** 2024-09-11

**Authors:** Shenjie Wu, Randy W. Schekman

**Affiliations:** Department of Molecular and Cell Biology, Howard Hughes Medical Institute, University of California, Berkeley, Berkeley, CA, United States

**Keywords:** alpha synuclein, Parkinson’s disease, Braak hypothesis, unconventional secretion, receptor-mediated uptake, endosome escape, tunneling nanotube, extracellular vesicle (EV)

## Abstract

An emerging theme in Parkinson’s disease (PD) is the propagation of α-synuclein pathology as the disease progresses. Research involving the injection of preformed α-synuclein fibrils (PFFs) in animal models has recapitulated the pathological spread observed in PD patients. At the cellular and molecular levels, this intercellular spread requires the translocation of α-synuclein across various membrane barriers. Recent studies have identified subcellular organelles and protein machineries that facilitate these processes. In this review, we discuss the proposed pathways for α-synuclein intercellular transmission, including unconventional secretion, receptor-mediated uptake, endosome escape and nanotube-mediated transfer. In addition, we advocate for a rigorous examination of the evidence for the localization of α-synuclein in extracellular vesicles.

## Introduction

The abnormal deposit of inclusion structures known as Lewy bodies (LBs) and Lewy neurites (LNs) in neurons and other neuronal cells is a hallmark of PD, dementia with Lewy bodies (DLB) and multiple system atrophy (MSA). These inclusions may also be found to coincide in a range of other neurodegenerative diseases, including Alzheimer’s disease (AD) (Irwin et al., 2017). The association of LB with PD was first described by Dr. Frederich Lewy in 1912 through his study of the pathological anatomy of PD patients (Engelhardt and Gomes, 2017). These inclusion bodies were subsequently named after him. Microscopic studies have long revealed the filamentous structures within the LBs (Spillantini et al., 1998). Recent work has also identified lipid components, including damaged organelles and vesicles co-aggregated with the fibrillar core (Shahmoradian et al., 2019). It took over 80 years after the initial discovery of LBs for the key component in their formation their major component to be identified, eventually by immunostaining—a small synaptic protein named α-synuclein (Spillantini et al., 1997).

The synuclein family of proteins comprises three members: α, β and γ-synuclein. The name “synuclein” reflects their predominant cellular localization in both the nucleus and presynaptic terminals. Compared to other synucleins, α-synuclein has a higher propensity to misfold and form aggregates. In its aggregated form, α-synuclein creates fibrillar structures, as observed inside LBs. Several mutations and a triplication of *SNCA*, the gene encoding α-synuclein, are linked to the early onset of PD (Ozansoy and Basak, 2013). Together, biochemical and genetic evidence lead to a hypothesis that the pathogenic transition of soluble α-synuclein into fibrillar inclusions inside LBs is a key event in PD pathogenesis. Numerous therapeutic efforts have been made to cure PD by clearing or disaggregating α-synuclein, yet little progress has been achieved in this direction (Fields et al., 2019).

As a movement disorder, PD is characterized by the degeneration of dopaminergic neurons in the substantia nigra (Chinta and Andersen, 2005). However, LBs are not confined to the substantia nigra but can also be found in various brain regions, depending on the disease stage. The spatial and temporal distribution of LB pathology supports a prion hypothesis of α-synuclein, in which pathological α-synuclein propagates from one neuron to another, spreading toxicity as the disease progresses. Unlike cell-surface-anchored prions, α-synuclein is predominantly intracellular and lacks a classic signal peptide for secretion. How does α-synuclein transmit between cells? In recent years, multiple pathways have been proposed to explain potential routes of the intercellular transfer of α-synuclein. In this review, we will survey the experimental evidence for each pathway, particularly the hypothetical exosome-mediated α-synuclein transmission.

### Braak staging of PD

Progressive loss of specific dopaminergic neurons in the substantia nigra and the resulting motor disorders are considered the primary hallmarks of PD. However, clinical observations reveal that a series of non-motor symptoms, including loss of smell, sleep disorders and mood changes, often occur before the classical PD symptoms such as tremor, postural rigidity, and bradykinesia (Kumaresan and Khan, 2021). For example, hyposmia is associated with 90% of early-stage PD but is usually under-recognized when it occurs years or even decades before the first PD diagnosis (Xiao et al., 2014). Pathology involving LBs can also be found outside the substantia nigra; notably, the olfactory bulb is affected in 94.8% of PD cases (Beach et al., 2009). The pre-symptomatic PD pathology beyond the substantia nigra raises questions about the origin of the disease.

**Figure 1:**
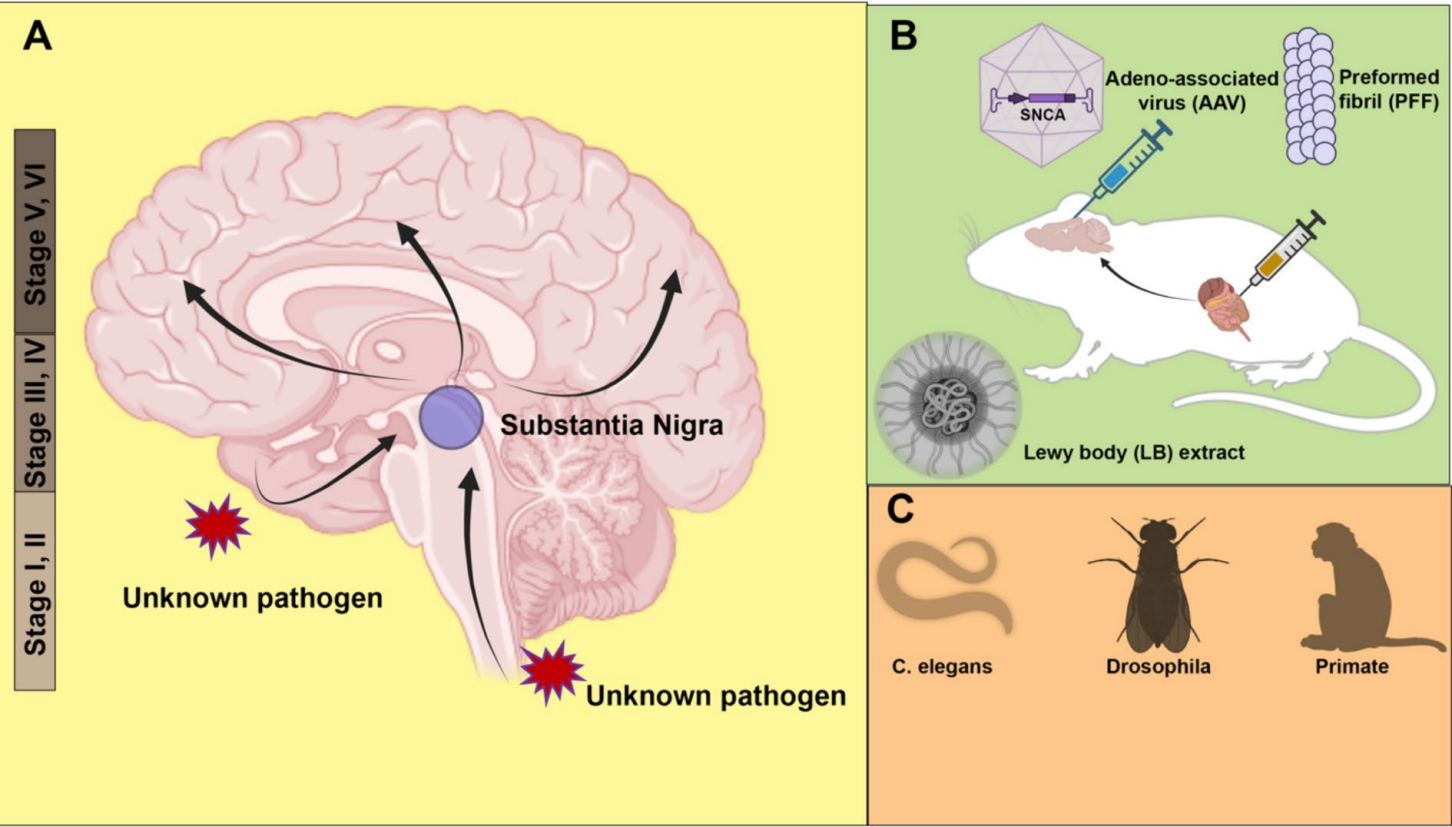
Animal models of α-synuclein transmission. **(A)** Braak staging of Parkinson’s disease (PD) topographic progression. According to the hypothesis, the origin of PD may start with the invasion by unknown pathogens via nasal or gastrointestinal peripheral system into the central nervous system. The pathology spreads from the olfactory bulb and lower brainstem to the substantia nigra of the midbrain, and further to the cortex. **(B)** Modeling pathology spread in rodent models. α-synuclein is introduced to animals in the form of adeno-associated virus (AAV), preformed fibril (PFF) or PD patient Lewy bodies extract. Both direct injection into the brain and peripheral tissues such as the gut cause the spread of pathology. **(C)** Other animal models, including roundworm (*Caenorhabditis elegans*), fruit fly (*Drosophila*) and non-human primates (Monkey) have also been used to model PD pathology. Created with BioRender.com.

Based on a study of more than a hundred autopsy brains, Braak and his team classified PD into six stages, with pathology assuming an upward trend in progression (Figure 1A) (Braak et al., 2003a). In the early stages (Stages I and II), LB pathology is confined to the dorsal motor nucleus of the vagus nerve, situated in the medulla oblongata of the brainstem and the anterior olfactory nucleus. Notably, in these stages, the substantia nigra, typically associated with PD pathology, remains unaffected. Subsequent stages (Stages III and IV), characterized by typical PD-related motor symptoms, involve neurons in the pars compacta of the substantia nigra. Especially at stage IV, marked damage to the melanin-containing neurons in the substantia nigra is observed. At the same time, damage accumulates in regions affected in the previous stages. As the disease continues to develop (Stages V and VI), pathology extends to neocortical territories. Cognitive dysfunction in patients is frequently observed at these final stages of PD.

There are two possible explanations for the Braak staging scheme: vulnerability versus spreading. The vulnerability theory suggests that the onset of pathology occurs simultaneously at multiple regions of the brain, with certain types of neurons more susceptible at different stages. In contrast, the spreading theory postulates an ascending route for transmission of the pathology from the peripheral to the middle brain and finally cortex. Braak himself hypothesizes that an unknown neurotropic pathogen enters through the enteric nervous system and traverses along the vagus nerve into the central nervous system (Braak et al., 2003b).

There is evidence both supporting and contradicting Braak-type spreading (Surmeier et al., 2017). Constipation and other gastrointestinal dysfunctions are risk factors for PD, suggesting the disease may originate from the gut. In fact, viral and bacterial infections have been proposed to directly or indirectly lead to PD, suggesting the existence of an inducing pathogen (Leta et al., 2022). Grafted fetal dopaminergic neurons in PD patients have shown a certain chance of developing LBs after transplantation, suggesting a potential transfer of LBs (Kordower et al., 2008; Li et al., 2008). In contrast, all vulnerable neurons share physiological features, including long and branched axons, high levels of α-synuclein expression, and elevated oxidative stress. A network modeling study showed that the pattern of LB pathology matches the anatomical connectivity and the endogenous expression of α-synuclein (Henderson et al., 2019). A transcriptomic study found conserved gene and pathway changes in cells that are vulnerable to LB pathology in mice and PD patients (Goralski et al., 2024). It is plausible that selective vulnerability and the putative transmission together contribute to the Braak staging of PD (Henderson et al., 2022).

### Animal models of α-synuclein transmission

A strong argument supporting the spreading of α-synuclein comes from animal model studies, especially in mice. Various α-synuclein spreading models have been developed to recapitulate the Braak staging in human PD patients. Based on the host-to-graft transmission observed in transplantation surgery, one study found that injected green fluorescent protein (GFP)-labeled mouse cortical neuronal stem cells developed α-synuclein pathology in a time-dependent manner (Desplats et al., 2009). Adeno-associated virus (AAV) expression has also been used as a tool to model PD in mice (Figure 1B). The low immunogenicity and the ability to transduce neurons make AAV an ideal vector for delivering human α-synuclein to be expressed in the rodent brain. In many mouse models involving AAV injection, the pathology spread beyond the injection sites, causing loss of tyrosine hydroxylase-positive dopaminergic neurons (Huntington and Srinivasan, 2021).

The most widely used PD animal model is the α-synuclein preformed fibril (PFF) injection model (Figure 1B). *In vitro*, purified α-synuclein forms fibrillar aggregates upon rigorous agitation. Cryo-electron microscopy (cryo-EM) structures of α-synuclein fibrils reveal a rich β-strand structure in a twisted protofilament (Guerrero-Ferreira et al., 2020; Yang et al., 2022). It is worth noting that a diversity of PFF structures may be associated with different types of synucleinopathies (Yang et al., 2022; Schweighauser et al., 2020; Strohaker et al., 2019). A recently developed seed amplification assay (SAA) claims to generate conformationally specific PFFs based on the initial seed input (Concha-Marambio et al., 2023). Such an assay corroborates the prion-like properties of PFFs. A digital version of SAA has been implemented for sensitive and quantitative amplification of endogenous α-synuclein aggregates (Gilboa et al., 2024).

Following intrastriatal inoculation of PFF in mice, LB pathology accumulates and spreads in a stereotypical manner, notably leading to dopaminergic neuron loss and motor deficits (Luk et al., 2012). The injection of PFF can induce LB-like pathology, including accumulation of insoluble phosphorylated α-synuclein at serine 129. In addition to mouse models, similar pathology accumulation and spread have been observed in PFF injection in other animals, including rats (Paumier et al., 2015) and non-human primates (Shimozawa et al., 2017). Consistent with the Braak hypothesis, PFF injection into the olfactory bulb (Rey et al., 2018) and peripheral tissues, including the gut (Kim et al., 2019), also causes pathology propagation. Gut-to-brain transmission was found to be specific to aged mice (Challis et al., 2020). Non-α-synuclein fibrils, such as bacterial curli amyloid, also promote α-synuclein aggregates in the gut and brain, suggesting a cross-seeding mechanism for transmission (Sampson et al., 2020). The same curli protein has been identified as a promoting factor for α-synuclein aggregation in *Caenorhabditis* elegans (Wang et al., 2021).

Although there are still reproducibility issues with the PFF injection model, it remains a powerful and popular tool to model PD pathology *in vivo*. Quality control measures, including conformation validation, fibril length control by sonication, and storage conditions, are all important considerations for improving reproducibility (Polinski et al., 2018). Compared with other animal models, PFF injection allows examination of pathology formation and transmission without overproduction of α-synuclein and enables spatially precise inoculation with a relatively small amount of PFF. In a recent work, injection of aggregates amplified from a LB extract, rather than conventional PFF, induces pathology that more faithfully recapitulating the LB pathology in PD patients (Uemura et al., 2023) (Figure 1B). In the future, more physiologically relevant animal models, such as animals with endogenously aggregating α-synuclein, may provide better insights into the pathogenesis of PD. In addition to rodent models, simple organisms such as *Caenorhabditis* elegans (Chen M. et al., 2022) and *Drosophila* (Feany and Bender, 2000) have also been used to model PD with exogenous expression of human α-synuclein (Figure 1C). The recapitulation of pathology transmission in various animal models supports the hypothesis of cell-to-cell transmission of α-synuclein.

### Cellular models of α-synuclein transmission

Despite not being a purely genetic disease, genetic mutations in several genes cause or increase the risk of PD. These PD genes, including *SNCA*, *LRRK2*, *PRKN*, and *PINK1*, provide opportunities for understanding the molecular and cellular basis of PD pathogenesis. Compared with the animal models discussed in the previous section, cellular models are more suitable for identifying genes and proteins involved in transmission, and for large-scale drug or genetic screening targeting the different steps of transmission. We will discuss these genes in the following sections of this review.

Single-cell organisms such as yeast and bacteria have been used to study the conserved functions of α-synuclein and to serve as expression vehicles for the protein production of α-synuclein. Yeast, a powerful tool in classic genetics and cell biology, has been utilized to uncover cellular functions of α-synuclein, including a role in intracellular trafficking (Gitler et al., 2008), mitochondria-mediated toxicity (Buttner et al., 2008), and recently as an RNA-binding protein (Hallacli et al., 2022). Interestingly, α-synuclein expressed in *Escherichia coli* is secreted into the periplasmic space (Ren et al., 2007). Studies in such simple cellular systems reveal a potential role for α-synuclein in the protein trafficking pathway.

**Figure 2:**
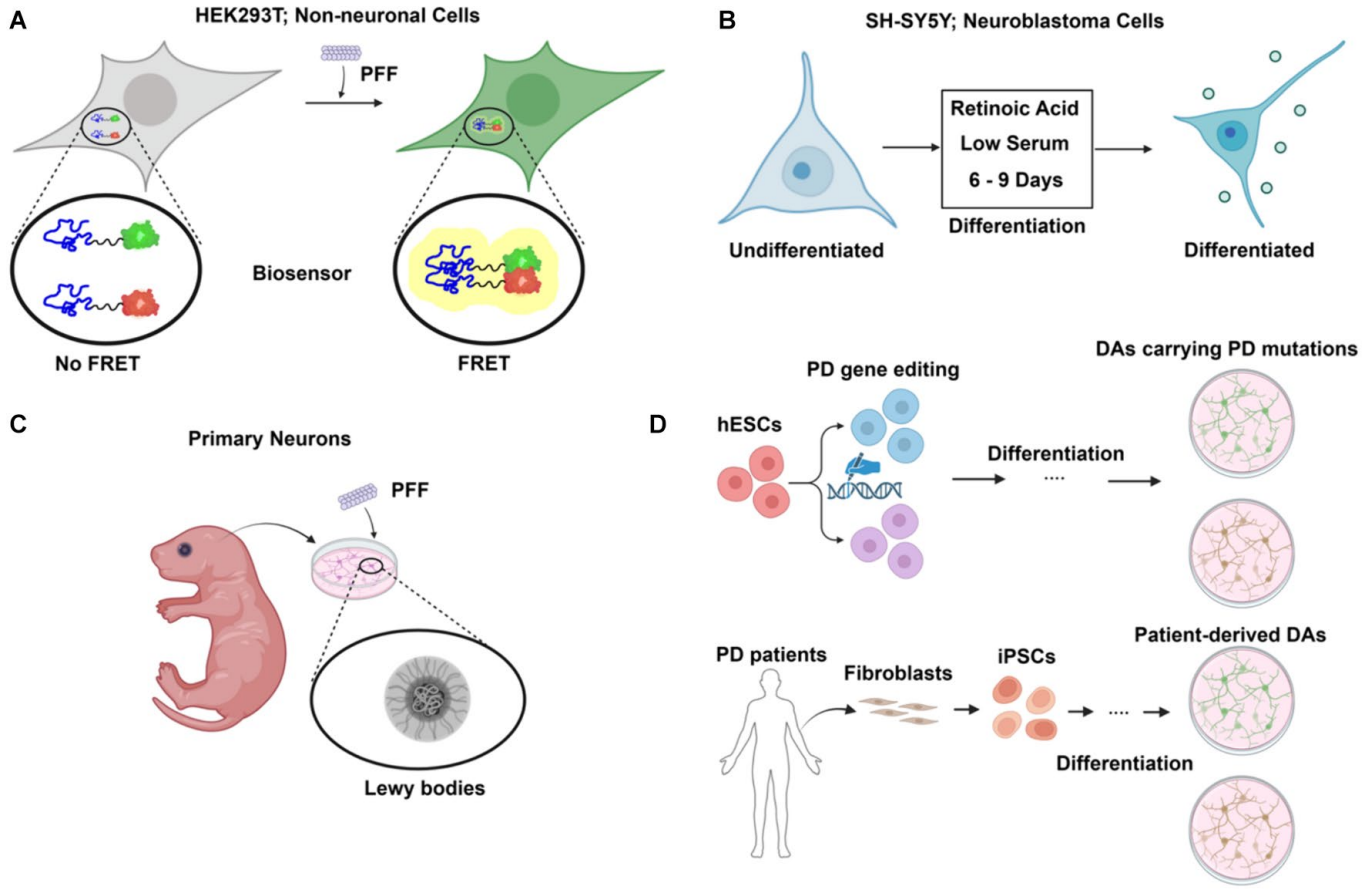
Cellular models of α-synuclein pathology in Parkinson’s disease (PD). **(A)** FRET-based biosensor assay. Established primarily in non-neuronal cells such as HEK293T, the biosensor assay allows sensitive and quantitative detection of aggregate formation upon preformed fibril (PFF) challenge. **(B)** Differentiated neuroblastoma cell as PD model. Using a simple differentiation scheme by treatment of retinoic acid (RA) and low serum for 6–9 days, neuroblastoma cell lines such as SH-SY5Y can express dopaminergic neuronal markers. **(C)** Primary neurons from rodent models. Primary neurons are isolated and challenged with PFF, leading to the formation of Lewy body-like structures. **(D)** Human stem cell-derived models for PD. Human embryonic stem cells (hESCs) are edited to carry PD mutations and differentiated into dopaminergic neurons (DAs), providing a model to study PD-specific genetic alterations. Additionally, fibroblasts from PD patients are reprogrammed into induced pluripotent stem cells (iPSCs) and differentiated into dopaminergic neurons, providing a patient-specific model to investigate PD pathology. Created with BioRender.com.

Non-neuronal, immortalized mammalian cell lines, e.g., HEK293, are easy to maintain and scale up for biochemical studies. Ectopic expression of α-synuclein is usually required because endogenous levels in these cell lines are much lower than in neuronal cells. A fluorescence resonance energy transfer (FRET)-based biosensor reporter system was first developed in the HEK293 cell line for the detection of intracellular aggregate formation (Maina et al., 2023) (Figure 2A). Exogenous fibrillar seeds are internalized by the cells and following escape into the cytoplasm, the formation of aggregates with tagged α-synuclein is monitored by fluorescence imaging and flow cytometry. CRISPR-Cas9 based genetic screening and biochemical studies of aggregate formation have been carried out using the biosensor (See et al., 2021). However, there is also a challenge in determining if the FRET signal observed in biosensor experiments represents actual fibril assembly (Kaniyappan et al., 2020).

Dopaminergic neuron (DA)-like cells can be generated from neuroblastoma cell lines such as SH-SY5Y and SK-N-MC (Figure 2B). A combination of serum reduction and the addition of retinoic acid (RA) or other hormones upregulates neuronal, especially DA-neuron markers expression (Korecka et al., 2013). Differentiated SH-SY5Y cells highly express endogenous α-synuclein and are susceptible to extracellular α-synuclein-induced toxicity (Emadi et al., 2009). However, as an immortalized cell line with cancer features, SH-SY5Y cells have altered metabolism and are not phenotypically DA-like even after differentiation.

Primary neuronal culture offers an alternative to avoid the genetic changes present in neuroblastoma cells (Figure 2C). The addition of PFF to primary neurons from mice recruits endogenous α-synuclein to form aggregates that are phosphorylated and detergent-insoluble, as in LBs (Volpicelli-Daley et al., 2014). Prolonged incubation of PFF with the primary neuronal culture for up to 21 days reveals a structural and morphological transition of α-synuclein aggregates into a state that closely resembles LBs in patients (Mahul-Mellier et al., 2020). Internalization and intercellular transfer of PFF have also been observed in primary neurons cultured in microfluidic devices (Freundt et al., 2012).

Embryonic stem cells (ESCs), derived from early-stage embryos, are both self-renewable and have the potential to develop into various specialized cell types, including the DAs affected in PD (Piao et al., 2021) (Figure 2D). This provides a unique opportunity to study PD in a physiology-relevant cellular system. Moreover, CRISPR-Cas9 based genome editing tools such as prime editing have been applied and optimized for human stem cells to allow highly efficient, large-scale modeling of roles of PD genes (Li et al., 2022). However, the embryonic origin of ESCs raises ethical concerns. Induced pluripotent stem cells (iPSCs), reprogrammed to a pluripotent state from somatic cells, share the same genetic background as the donors (Figure 2D). Robust protocols have been established to generate DAs using PD patient skin fibroblasts (Fernandes et al., 2016). Patient-derived DA cultures allow for functional study in the context of the complex genetic risk factors of PD in a personalized setting. Stem cells can also be used to generate 3D midbrain-like organoids, which present a more biologically relevant cellular system than traditional 2D cell culture (McComish et al., 2022). Such an effort provides an important resource in the use of stem cells to study PD.

### Unconventional secretion of α-synuclein

Besides being a primarily intracellular protein, α-synuclein is also found in various extracellular fluids, such as cerebrospinal fluid (CSF) and blood plasma (El-Agnaf et al., 2003). While part of the extracellular presence of α-synuclein may be due to passive cell lysis, a question remains whether any extracellular α-synuclein is released actively and selectively from cells followed by spreading between cells. Unlike conventional secretory proteins, α-synuclein does not contain an amino-terminal signal peptide to target it to the endoplasmic reticulum (ER) for secretion. The process by which leaderless proteins reach the plasma membrane and extracellular space is collectively termed “unconventional secretion” (Zhang and Schekman, 2013). Unconventional protein secretion (UPS) comprises diverse protein trafficking pathways that bypass the classic ER-Golgi route mediated by COPII vesicles. The mechanisms of several unconventionally secreted cargoes, including fibroblast growth factor 2 (FGF2) and Interleukin-1 beta (IL1β), have been systematically studied (Pallotta and Nickel, 2020). It is worth noting that the export of α-synuclein has been shown to be independent of TMED10, which regulates IL1β secretion (Zhang M. et al., 2020).

The active release of α-synuclein has been observed in many *in vitro* cell culture systems. Lee et al. demonstrated that a small percentage of α-synuclein is rapidly secreted from differentiated SH-SY5Y cells in an ER-Golgi independent manner (Lee et al., 2005). Several factors, including autophagy (Poehler et al., 2014), calcium (Emmanouilidou et al., 2010), and inflammatory cytokines such as tumor necrosis factor α (TNF-α) (Bae et al., 2022), stimulate α-synuclein secretion. Stress induced by the inhibition of protein degradation by lysosomes and proteasomes also enhances α-synuclein secretion (Jang et al., 2010). Different forms of α-synuclein, including monomers, oligomers and aggregates, all respond to these signals for unconventional secretion. SNARE-dependent lysosomal exocytosis has been shown to mediate the release of aggregated forms of α-synuclein (Xie et al., 2022).

**Figure 3:**
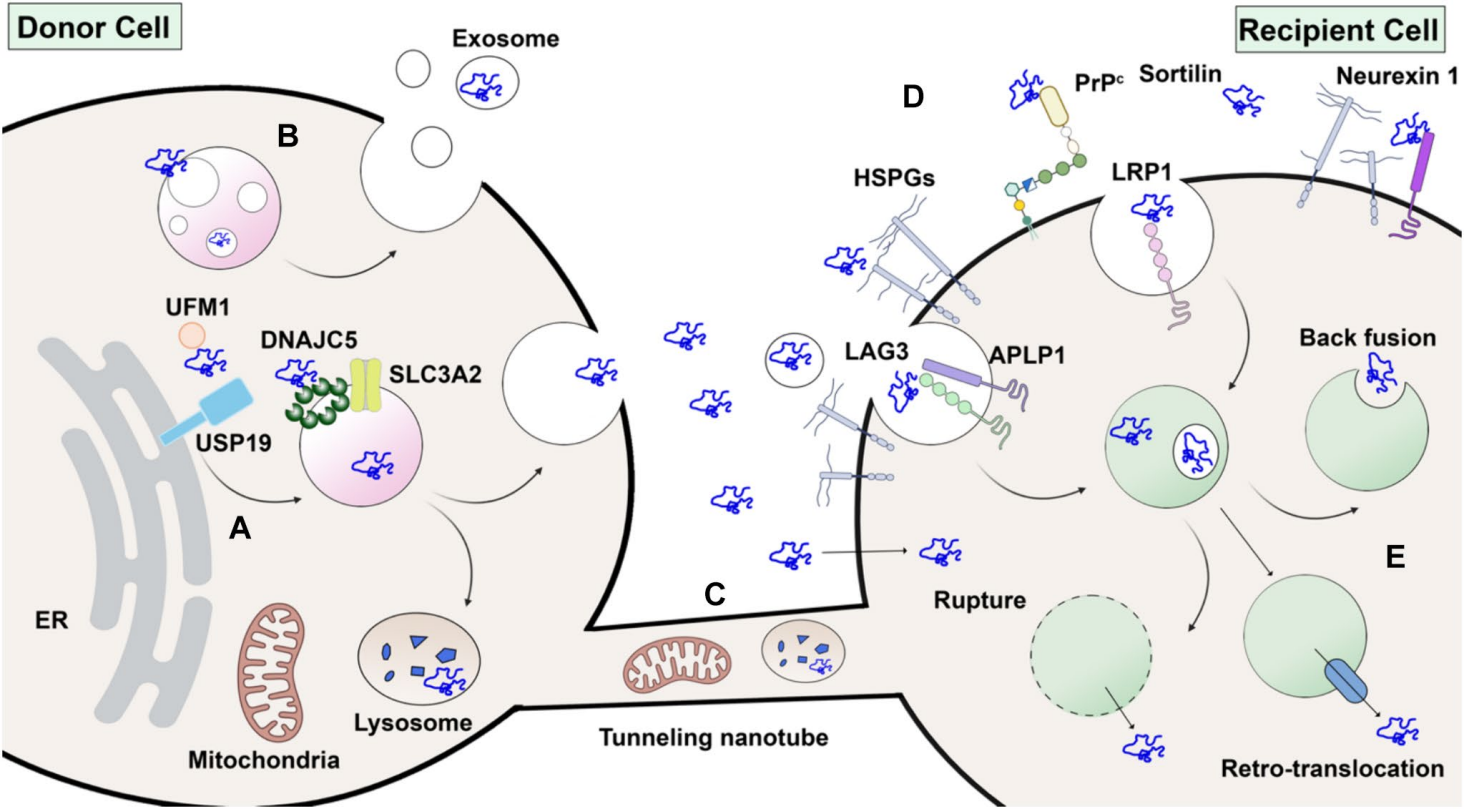
Without a signal peptide, α-synuclein may transfer from donor cell to recipient cell via several routes. **(A)** Unconventional secretion as naked proteins. α-synuclein modified by ubiquitin-fold modifier 1 (UFM1) is recognized by USP19 on the ER surface. Subsequently, α-synuclein is translocated into the lumen of endosomes or other organelles by membrane-anchored palmitoylated DNAJC5 oligomer. Peri-nuclear SLC3A2 is a reported interacting protein of DNAJC5. Upon fusion with the plasma membrane, α-synuclein is secreted without membrane enclosure. **(B)** Packaged inside exosomes. α-synuclein may also be internalized into the intraluminal vesicles (ILVs) of endosomes and secreted in exosomes. **(C)** Membrane tubular connection. α-synuclein, together with subcellular organelles such as mitochondria and lysosomes, may transfer through tunneling membrane tubes directly. **(D)** Direct penetration across plasma membranes or receptor-mediated endocytosis in recipient cells. HSPG, LAG3, APLP1, LRP1, PrP^C, Sortilin and Neurexin 1 are potential candidates for receptors of extracellular α-synuclein. **(E)** Once endocytosed, α-synuclein may escape from endosomes by membrane rupture, retro-translocation or back fusion. Created with BioRender.com.

The misfolding-associated protein secretion (MAPS) pathway is a recently discovered mechanism that mediates the secretion of several misfolding-prone proteins, including α-synuclein (Figure 3A). In MAPS, an ER-localized deubiquitylase, USP19, recruits misfolded protein cargos via its chaperone activity and leads to their secretion via late endosomes (Lee et al., 2016). Another HSC70 co-chaperone protein, DNAJC5, has been reported to regulate α-synuclein secretion (Fontaine et al., 2016). Besides α-synuclein, DNAJC5 is also responsible for the release of many other neurodegenerative disease-associated proteins, such as TDP-43 and tau. A later study found that DNAJC5 seems to function downstream of USP19 in MAPS (Xu et al., 2018). DNAJC5 has been shown to be extensively palmitoylated and associated with endosomes, where α-synuclein translocation occurs in an unfolding-independent manner (Wu et al., 2023). Genetic sequencing of a patient identified with Parkinsonism symptoms revealed an L116 deletion in the DNAJC5 gene (Guo et al., 2024). The L116 deletion in DNAJC5 has also been linked to an aberrant pattern of palmitoylation. SLC3A2, known as a component of an amino acid transporter complex, was found to interact with DNAJC5 and regulate perinuclear localization of DNAJC5 and α-synuclein secretion (Lee et al., 2023). Notably, α-synuclein secretion promoted by either USP19 or DNAJC5 is mostly soluble.

α-synuclein undergoes multiple post-translational modifications (PTMs) at various sites. Phosphorylation at serine 129 is widely used as a marker of α-synuclein aggregates and has been shown to increase in LBs in PD patients (Fujiwara et al., 2002). Notably, a portion of these α-synuclein aggregates is truncated (Sorrentino and Giasson, 2020) and nitrated (Giasson et al., 2000). Other PTMs also affect the aggregation propensity of α-synuclein and may influence its secretion and spread. Modification by a ubiquitin-like protein, ubiquitin-fold modifier 1 (UFM1), has recently been shown to enhance α-synuclein secretion dependent on ER-localized USP19 (Wang et al., 2024). Although *in vitro* synthesized glycosylated α-synuclein has been shown to be protected from aggregation (Lewis et al., 2017), there is no evidence that α-synuclein is translocated into the ER lumen and glycosylated *in vivo*. The preponderance of evidence supports a model of α-synuclein secretion independent of ER-Golgi transit.

### Receptors for α-synuclein

Once secreted into the extracellular space, intercellular transfer of α-synuclein would require an endocytic process or some means of direct transfer across the plasma membrane of a neighboring cell. α-synuclein itself has intrinsic lipid-binding affinity (Jo et al., 2000). Upon binding to lipid, α-synuclein adopts an α-helical conformation and oligomerizes (Burre et al., 2014; Schwarz et al., 2023). The N-terminal repeats of α-synuclein have been shown to promote efficient direct translocation into cells independent of endocytosis (Ahn et al., 2006). Other studies suggest that α-synuclein enters cells via receptor-mediated endocytosis, for which several different receptors have been suggested (Figure 3D). PFFs have been widely used in these studies. Heparan sulfate proteoglycans (HSPGs) are highly glycosylated proteins located on the cell surface and act as general receptors for many macromolecular cargoes (Christianson and Belting, 2014). Viruses and exosomes attach to and interact with HSPGs during their internalization. HSPGs were also found to play a role in PFF internalization (Zhang Q. et al., 2020).

In addition to HSPGs, other cell surface proteins have been reported to be co-receptors for α-synuclein internalization. A recent study using expression cloning in SH-SY5Y cells identified lymphocyte activation 3 (LAG3) as a PFF-specific cell surface binding protein (Mao et al., 2016). LAG3 belongs to the immunoglobulin family, typically expressed in immune cells, and its role as an immune checkpoint has been widely studied. In this study, deletion of LAG3 reduced α-synuclein endocytosis and pathology both *in vitro* and *in vivo*. Later biophysical studies revealed the potential charged binding interface of PFF on LAG3 and further suggested that the pathology-related serine 129 phosphorylation on α-synuclein enhanced its binding affinity (Zhang et al., 2021). Another transmembrane protein, amyloid β precursor-like protein 1 (APLP1), was found to be involved in the internalization of α-synuclein by interaction with LAG3 (Mao et al., 2024). However, these results are of questionable significance as another study reported that LAG3 is not expressed in various neuronal cells (Emmenegger et al., 2021). Another potential receptor, glycosylated neurexin 1, was found in a screen and was later demonstrated to bind to acetylated α-synuclein (Birol et al., 2019; Birol et al., 2023).

Low-density lipoprotein receptor-related protein 1 (LRP1) belongs to the low-density lipoprotein receptor (LDLR) family, which has been demonstrated to participate, together with HSPG, in mediating both Aβ (Kanekiyo et al., 2011) and tau (Rauch et al., 2020) uptake in Alzheimer’s disease. Recently, LRP1 was found to mediate the uptake of monomeric, oligomeric, and fibrillar α-synuclein (Chen K. et al., 2022). This indicates a conserved mechanism and shared receptors for the uptake and spread of different neurodegenerative disease-related proteins. Other membrane proteins, such as cellular prion protein (PrP^C^) (Ferreira et al., 2017) and sortilin (Ishiyama et al., 2023), have also been found to interact with α-synuclein oligomers and fibrils, respectively. Since there is still debate about the toxicity of different forms of α-synuclein, it remains to be seen which receptors play a more disease-relevant role during the spread of α-synuclein pathology and disease progression. The expression profile of each proposed receptor in dopaminergic and other brain cells will also inform the transmission route and vulnerability of different cell types.

### Endosome escape of α-synuclein

Most endocytic cargoes end up in lysosomes and are degraded, and the resulting metabolites are recycled in the recipient cells. However, endocytic α-synuclein can escape this degradation fate and be delivered into the cytoplasm of the recipient cells for continuous propagation. The phenomenon of endosome escape is known for various cell-penetrating peptides, viruses, lipid nanoparticles (LNPs), and arguably exosomes. Endosome escape may be mediated either by membrane fusion or disruption. For enveloped viruses, such as influenza A virus, low pH triggers a conformational change in the hemagglutinin, exposing its fusion peptide to catalyze membrane fusion and the cytoplasmic delivery of its genetic content (Ivanovic et al., 2013). In the absence of a fusogen, as with LNPs, the interaction between ionized lipids from LNPs and the endosomal luminal membrane or the proton sponge effect of LNPs, causing swelling and bursting of endosomes, releases the cargo (Chatterjee et al., 2024).

To escape from endo/lysosomes, α-synuclein must survive in a proteolytic environment or escape before the acidification and maturation of the lysosome (Figure 3E). Many lysosomal proteases, including cathepsin B and L, can degrade α-synuclein fibrils into fragments (McGlinchey and Lee, 2015). However, studies have found that internalized α-synuclein fibrils are poorly degraded, perhaps by inhibiting autophagy (Tanik et al., 2013). In fact, α-synuclein fibrils internalized by primary neuronal cell cultures may transform over time into lipid-rich aggregates containing vesicles and damaged organelles that mimic Lewy bodies (Mahul-Mellier et al., 2020). Escape from the endosome may require the action of a PIKfyve kinase. An inhibitor of this enzyme appears to block trafficking of endocytic α-synuclein, which accumulates in an early endosome, suggesting escape may occur prior to reaching the lysosome in the endocytic pathway (See et al., 2021).

*In vitro*-generated α-synuclein internalized into neuronal cells has been reported to induce lysosomal rupture (Freeman et al., 2013). Lipofectamine, a cationic-lipid transfection reagent, has been widely used to enhance the delivery of α-synuclein aggregates and cause endosome rupture (Luk et al., 2009). These studies suggest that endo/lysosomal rupture, caused either directly by α-synuclein high-order assembly or other factors, may contribute to the escape of α-synuclein (Jiang et al., 2017). The ability to cause endosome rupture may be a conserved escape mechanism for internalized protein aggregates (Luk et al., 2009). Supporting this idea, autophagy of damaged lysosomes, or lysophagy, has been shown to protect against α-synuclein escape and propagation (Kakuda et al., 2024).

It is plausible that α-synuclein may also use other membrane penetration mechanisms to escape from intact endo/lysosomes or even directly translocate across the plasma membrane. One report documented an intermediate state of partially inserted fibril on the lipid membrane (Fusco et al., 2017). Another group found that incubation at 4°C or treatment with endocytosis inhibitors did not affect α-synuclein internalization (Ahn et al., 2006). A recent study found that the endosomes of neurons have pores, regulated by PIKfyve kinase, through which internalized PFFs pass to engage cytoplasmic α-synuclein in further aggregation and compromised cell viability (Sanyal et al., 2024). Given that folding-stable protein fusions of α-synuclein can translocate from the cytosol to the lumen of the endosome as a prelude to unconventional secretion (Wu et al., 2023), it will be interesting to consider how pores may form and permit bidirectional but selective endosomal membrane permeation. Understanding the multifaceted endosome escape mechanisms of α-synuclein may inform future therapeutic interventions.

### Nanotube-mediated transfer of α-synuclein

In addition to vesicular and non-vesicular paracrine signaling between cells, biochemical cargo can also be transported intercellularly by tunneling tubular connections based on direct cell-to-cell contact. Unlike tight junctions or synapses between cells, tunneling nanotubes (TNTs) are open-ended and thus mediate direct cytoplasmic cargo exchange (Cordero Cervantes and Zurzolo, 2021). Many biomolecules and subcellular organelles, including RNA, proteins, virus particles, lysosomes, and mitochondria, have been reported to transfer between cells via open-ended TNTs. The TNT structure dynamically undergoes formation, remodeling, and detachment, establishing an *ad hoc* targeted cargo transfer in neighboring cells. The diameter of TNTs varies from ~100 nm to microns in width. Micrometer-length TNT structures have been observed in cell culture. Fusogenic proteins involved in cell-to-cell or virus-to-cell fusion may participate in the formation of open-ended connections in TNTs (Zhang and Schekman, 2023).

The Zurzolo group showed that PFFs are transferred between neurons via a cell-to-cell contact mechanism (Abounit et al., 2016). Morphologically, lysosomes containing PFFs were observed in a TNT-like structure formed between cells (Figure 3C). In subsequent work using light and electron microscopy, this group suggested that lysosomes containing PFFs were remodeled both morphologically and functionally to be hijacked as a Trojan horse to spread α-synuclein via TNTs (Dilsizoglu Senol et al., 2021).

Mitochondria are another organelle susceptible to α-synuclein pathology (Figure 3C). Microglia, the brain-resident immune cells, are responsible for the clearance of extracellular debris, including α-synuclein aggregates. A recent study found that upon PFF challenge, microglia are activated and form a cellular network connected by TNTs. Both α-synuclein aggregates and mitochondria were found redistributed among connected microglia, alleviating the cytotoxicity burden of affected cells (Scheiblich et al., 2021). Similar TNT-mediated transfer of mitochondria and α-synuclein has been found between microglia and neuronal cells (Chakraborty et al., 2023). Transfer of α-synuclein has also been observed in other brain cell types, including microvascular endothelial cells (Duan et al., 2023), pericytes (Dieriks et al., 2017), and astrocytes (Rostami et al., 2017). As the mechanistic studies of genes and proteins involved in the formation and regulation of TNTs is still evolving, it remains to be seen if any of these TNT-related genes play a role in the spread of α-synuclein pathology *in vivo*.

### Exosomes: do they play a role?

Extracellular vesicles (EVs) are a diverse group of membrane-bound vesicles derived from the direct budding of the plasma membrane or intra-luminal vesicles from endosomes (van Niel et al., 2018). Exosomes are defined as small EVs that bud into endosomes to form the multivesicular body and then are discharged when the multivesicular body (MVB) fuses with the plasma membrane (Kalluri and LeBleu, 2020). Cytoplasmic proteins and small RNA molecules are packaged into exosomes, sometimes by a selective mechanism, especially for miRNA (Qiu et al., 2021). Much interest in exosome research focuses on their potential role as a signaling mechanism between cells, enabling functional delivery of biomolecules into recipient cells. Thus, exosomes can be yet another vehicle for α-synuclein transmission in PD and used as a biomarker for diagnosis (Figure 3B) (Gualerzi et al., 2024).

**Figure 4:**
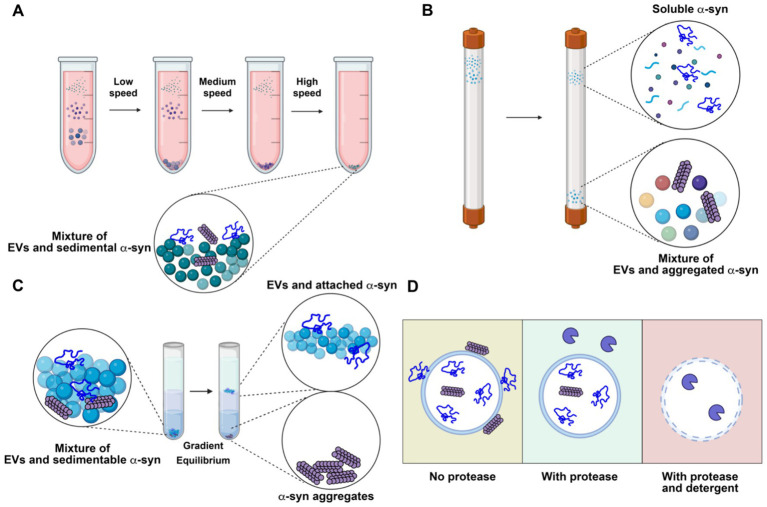
Representative analytical methods for characterizing extracellular vesicles (EVs) and their limitations in resolving the localization of α-synuclein (α-syn). **(A)** Differential centrifugation. The sample is subjected to serial low, medium and high-speed centrifugation. α-synuclein aggregates remain with concentrated EVs. **(B)** Size exclusion chromatography. Molecules in the sample are separated by their sizes. Soluble α-synuclein is purified from EVs, while aggregate α-synuclein remains with purified EVs. **(C)** Density gradient centrifugation. The high-speed pellet from differential centrifugation is resuspended in a high density medium and then overlaid with a gradient and centrifuged until buoyant equilibrium is reached. High-density α-synuclein is purified from buoyant EVs. Membrane-associated α-synuclein remains with purified EVs. **(D)** Protease protection assay. Without protease treatment, α-synuclein remains intact, regardless of its topology (left). With protease treatment, surface-exposed α-synuclein is degraded, while membrane-enclosed α-synuclein remains intact (middle). With both protease and detergent treatment, α-synuclein inside EVs is exposed and degraded (right). Created with BioRender.com.

Many studies have reported that α-synuclein can be secreted via exosomes (Emmanouilidou et al., 2010; Jang et al., 2010; Harischandra et al., 2019). However, these studies often face challenges due to inadequate purification methods, as thoroughly discussed in our review on EV functions in cancer (Shurtleff et al., 2018). The commonly adopted methods for EV isolation include precipitation by crowding reagents, size exclusion chromatography, differential centrifugation, and density gradient centrifugation, each with its own strengths and limitations. Crowding reagents and differential centrifugation allow rapid, easy enrichment of EVs from biological samples. However, large non-vesicular particles co-precipitate with crowding reagents or co-sediment with EVs by differential centrifugation (Figure 4A). Size-exclusion chromatography effectively separates EVs from small, soluble molecules, but large aggregates may elute in the same fraction with EVs (Figure 4B). Density gradient centrifugation is the only method capable of removing non-buoyant large aggregates from buoyant EVs and can further separate different populations of EVs based on their buoyant density. On the downside, density gradient requires more time-consuming preparation, as achieving density equilibrium may take overnight centrifugation (Figure 4C).

An important consideration when examining potential EV cargo, including α-synuclein, is topology — whether the cargo is packaged inside EVs or merely bound to the membrane surface. Enzymatic treatment can digest and remove the surface-bound contaminants from membrane-protected cargoes (Figure 4D). Immuno-electron microscopy can confirm the morphology of the purified EVs and topology of the putative cargoes. In most studies with the conclusion of EV-bound α-synuclein, the evidence of α-synuclein in exosomes is primarily based on examination of high-speed pellets from precipitation by crowding reagent or differential centrifugation. Caution should be exercised when interpreting the data, as α-synuclein is an aggregation-prone protein that may co-sediment as large aggregates. Very few studies have examined the topology of α-synuclein using a protease protection assay. In our study, we found that α-synuclein is secreted into conditioned medium as a largely soluble, protease accessible, monomeric species with a small sedimentable fraction. Further, this material is non-buoyant, suggesting it exists in an aggregated, insoluble form rather than as *bona fide* membrane-enclosed cargo (Wu et al., 2023). Many other studies have also found that α-synuclein is mainly secreted in a soluble form (Fernandes et al., 2016; El-Agnaf et al., 2003; Xie et al., 2022). These studies do not exclude the possibility that a minor fraction of α-synuclein can be packaged into exosomes or other types of EVs *in vivo* by certain cell types. Sensitive, rigorous assays should be applied to demonstrate that α-synuclein is buoyant, membrane-protected, and derived from an endosome origin.

In the context of α-synuclein pathology and intercellular transmission, exosomes have yet to be demonstrated as an effective vehicle. First, it remains unclear whether the putative packaging of α-synuclein in exosomes is passive or involves the selective enrichment of the toxic form of α-synuclein. Second, the functional delivery of the putative exosomal α-synuclein to the cytosol of recipient cells likely requires exosome-plasma membrane or exosome-endosome membrane fusion. Third, including a critical control, such as soluble α-synuclein, to compare the delivery efficiency between soluble and exosomal α-synuclein would provide a convincing argument for the active role of exosomes in promoting α-synuclein transmission.

## Conclusion

The clinical observation of toxic α-synuclein transmission in PD presents a unique opportunity to uncover a novel protein trafficking mechanism and identify potential intervention targets for a common neurodegenerative disease that currently lacks effective therapeutics. Over the years, many advances have been made in developing tools and models to study the α-synuclein transmission mechanism, including the PFF-injection mouse model and iPSC-derived cell culture systems. These tools have facilitated numerous studies on genes and proteins involved in various steps of the transmission, such as secretion regulation, receptor interaction, and endosome escape.

In the future, the systematic and complementary use of different tools will provide more insights into the role of intercellular transmission of α-synuclein in PD progression. While ectopic expression of α-synuclein and treatment with PFF in common cell cultures provide large quantities of material for biochemical dissection, understanding transmission in a cell-type-specific and disease-relevant context will require DA cell culture using iPSC technology. The basic biology revealed in cell culture systems can be further examined *in vivo* using various animal models. Here, the relatively smaller genomes of simple organisms, such as *Drosophila* and *Caenorhabditis elegans,* make them convenient for genetic studies with less concern about gene redundancy and compensation. On the other hand, rodent and primate models remain the most relevant systems for modeling complex features of intercellular α--synuclein transmission, including transfer between different brain cell types such as neurons and glial cells and gut-to-brain communication, as suggested by the Braak model. Team-based research is critical in adopting the various systems for the study of α-synuclein transmission.

Despite some controversial and contradictory findings, it is exciting to explore whether any identified genes and mechanisms are relevant to PD risk and progression. In particular, we have highlighted a few technical considerations regarding the role of exosomes in the transmission of α-synuclein and PD diagnosis. While multiple parallel mechanisms may contribute to the pathological spread of α-synuclein, a key question in the field is to clarify the toxic form of α-synuclein and the major route of transmission between cells. The basic biology of misfolded protein transmission may inform new therapies for PD.
